# Longitudinal associations between positive psychological factors and cardiovascular health

**DOI:** 10.3389/fcvm.2026.1821675

**Published:** 2026-07-31

**Authors:** Cathy Degroote, Roland von Känel, Claudia Zuccarella-Hackl, Livia Thomas, Hugo Saner, Roland Wiest, Petra H. Wirtz

**Affiliations:** 1 Biological Work and Health Psychology, University of Konstanz, Konstanz, Germany; 2 Department of Consultation-Liaison Psychiatry and Psychosomatic Medicine, University Hospital Zurich, University of Zurich, Zurich, Switzerland; 3 Department of Psychology, University of Bern, Bern, Switzerland; 4 Institute for Social and Preventive Medicine, University of Bern, Bern, Switzerland; 5 Support Center of Advanced Neuroimaging, Institute of Diagnostic and Interventional Neuroradiology, University Hospital Bern, University of Bern, Bern, Switzerland; 6 Centre for the Advanced Study of Collective Behaviour, University of Konstanz, Konstanz, Germany

**Keywords:** intermediate biological risk factors for CHD, life satisfaction, mindfulness, optimism, positive affect

## Abstract

**Background:**

The health promoting effects of positive psychological factors in cardiovascular disease (CVD) are gaining increasing attention. Key factors such as optimism, mindfulness, positive affect, and life satisfaction have been linked to a lower incidence of CVD. While these effects are often explained by favorable health behaviors, potential biological mechanisms are insufficiently explored.

**Objectives:**

We aimed to to investigate independent associations between positive psychological factors and prospective changes in intermediate biological coronary heart disease (CHD) risk factors in CHD-patients, hypertensives (HT), and normotensive controls (NT).

**Methods:**

At baseline, 200 men (CHD=86,HT = 61,NT = 53) completed questionnaires on positive psychological factors and had blood samples collected. As CHD-risk factors lipid profiles (TC/HDL-ratio), blood glucose (HbA1c), coagulation (fibrinogen, D-dimer), and inflammation markers (CRP, IL-6, TNF-α) were measured. At 3-year follow-up, blood sample were collected from 126 men (NT = 34,HT = 37,CHD=55).

**Results:**

At baseline, the groups differed in positive affect only [F(2,197) = 3.63, *p* = .028], with highest levels in NT and lowest levels in CHD. Prospectively, higher baseline positive affect (*p* = .026) and life satisfaction (*p* = .026) were associated with lower CHD-risk over time. Specifically, higher baseline positive affect predicted lower increase in coagulation markers (*p* = .010), particularly fibrinogen (ß=-0.37, *p* = .003), while higher baseline life satisfaction predicted lower increase in inflammation markers, especially IL-6 (ß=-.38, *p* = .005). Neither optimism nor mindfulness were linked to change in CHD-risk factors over time.

**Conclusion:**

Positive affect and life-satisfaction may benefit cardiovascular health by favorably affecting coagulation and inflammation, with potential implications for CHD prevention and intervention strategies.

## 1 Introduction

Cardiovascular diseases (CVD) comprise a group of diseases affecting the heart and blood vessels that are a major cause of premature death and disability worldwide (1). A highly prevalent type of CVD is coronary heart disease (CHD) (1), characterized by stenosis of coronary arteries with atherosclerosis as the primary pathological process (2). While negative psychosocial risk factors like stress, depression, and anxiety, can increase the risk of CHD (3), recent research highlights the potential protective effects of positive psychological factors on cardiovascular health (4–6). Based on longitudinal studies, major positive psychological factors associated with lower incidence of CVD in initially healthy participants, and/or of cardiovascular events in patients with established CVD, include *optimism* (5, 7), *positive affect* and *happiness* (5, 8), and *satisfaction with life* (9, 10). Optimism is a trait cognitive state marked by hope and confidence in positive future outcomes (5, 11). Positive affect, including happiness, reflects well-being and contentment, while life satisfaction refers to perceiveing life as meaningful and of high quality (12). Additionally, *mindfulness*, a present-focused, non-judgemental awareness, has been linked to cardiovascular health behaviors, such as non-smoking and high physical activity (5, 13). Notably, cardiovascular health behaviors have been proposed to play a role in mediation of the positive health effects of positive psychological factors (e.g., 5, 13–16).

Research has begun to elucidate the biological mechanisms underlying the cardiovascular benefits associated with these factors. Main intermediate biological risk factors linked to atherosclerotic diseases include elevated levels of blood lipids, chronic increase in blood glucose, coagulation markers, and inflammatory markers (17). Studies report both cross-sectional (18) and longitudinal associations between higher *optimism* and lower inflammation (19). Optimism is also associated with healthier blood lipid profiles cross-sectionally (20) and optimism training has been shown to reduce inflammation and coagulation [i.e., C-reactive protein (CRP) and fibrinogen] after 8 weeks (21). Moreover, higher *life satisfaction* has been associated with a lower incidence of diabetes (22), reduced cardiometabolic risk, evidenced by prospective assessments of blood lipid levels, glycosylated hemoglobin, and basal inflammation (CRP) in initially healthy participants (23, 24). *Positive affect* has been cross-sectionally associated with healthier lipid profiles in healthy participants (15), and with reduced inflammation (CRP, tumor necrosis factor-alpha (TNF-α), and interleukin (IL-6) in patients with heart failure (25). Moreover, prospective data in myocardial infarction patients further suggests higher positive affect to predict an improved blood lipid profile over time (26). However, no cross-sectional or prospective associations between *mindfulness* and intermediate biological risk factors have been reported to date.

From the available literature, it is unclear whether the reported associations between individual positive psychological factors and respective cardiovascular risk or outcome measures are independent. To the best of our knowledge, no studies have simultaneously assessed multiple positive psychological factors to test for independent associations with CVD endpoints or intermediate biological risk factors. We aimed to investigate the positive psychological factors—optimism, positive affect, life satisfaction, and mindfulness—simultaneously, assessing their independent associations with prospective changes in intermediate biological CVD risk factors, including blood lipids, glycosylated hemoglobin (HbA1c), coagulation and inflammation markers, over a 3-year follow-up. To ensure a high variation in risk factors, we recruited healthy normotensive (NT) controls, primarily unmedicated, otherwise healthy essential hypertensives (HT) as a high-risk group for CHD, and patients with manifest CHD. In a first descriptive step, our study aimed to compare positive psychological factors cross-sectionally among individuals with CHD, hypertension, and controls. Building on established prospective associations with CVD incidence and events (5, 7–9), we hypothesized that NT individuals would exhibit the highest levels of optimism, positive affect, and life satisfaction, with the lowest levels found in CHD-patients. Our primary aim was to explore the mechanisms underlying the clinical relevance of these positive psychological factors by examining whether they independently predict CHD risk in terms of prospective changes in markers of coagulation, inflammation, hyperlipidemia, and diabetes over a 3-year follow-up.

## 2 Materials & methods

### 2.1 Study participants

This investigation is part of a larger project exploring psychoneurobiological mechanisms associated with CVD (27) (Supplementary Material). The Ethics Committee of the Canton of Bern, Switzerland, approved the study protocol, which follows the Declaration of Helsinki. All participants provided written informed consent and received CHF20 for each assessment.

We recruited CHD-patients who had been discharged from the Bern University Hospital Cardiac Prevention and Rehabilitation Clinic at least six months before study participation. Additionally, we recruited apparently healthy, nonsmoking HT and NT of similar age with the assistance of the Swiss Red Cross of the Canton of Bern, following previously published methods [for further details see (27) and Suplemental-Material].

A two-step assessment procedure was implemented to classify blood pressure (BP, using Omron M6; Omron-Healthcare-Europe B.V., Hoofdorp, Netherlands) levels in non-CHD-participants as either normal or hypertensive. This included repeated measurements both at home and during study assessments. In accordance with the guidelines for home BP measurements, participants were categorized as hypertensive if their average home systolic BP (SBP) was ≥ 135mmHg and/or the average home diastolic BP (DBP) was ≥ 85mmHg (28) (see Supplementary Material). In the second step, the preliminary assignment to NT or HT respectively was verified by three additional BP measurements that were performed by trained personnel. These BP measurements were classified according to office BP cut-offs, with hypertension defined as mean study SBP of ≥ 140 mmHg and/or mean study DBP of ≥ 90 mmHg (29, 30). The final group assignment into the NT or HT groups was based on congruent home and study BP classification. To exclude potential cases with secondary hypertension, eligible HT provided blood samples for the routine assessment of serum creatinine, calcium, sodium, and potassium. No eligible HT was diagnosed with secondary hypertension.

A total of 200 men [*n*_NT_=53, *n*_HT_=61 (51 medication-free, 10 medicated), *n*_CHD_=86] met our inclusion criteria and filled out the questionnaires for the assessment of positive psychological factors at baseline. Of these, 126 [*n*_NT_=24, *n*_HT_=37 (30 medication-free, 7 medicated), *n*_CHD_=55] agreed to participate in the second assessment at follow-up 2.93 ± 0.05 (*SEM*) years later with 104 (*n*_NT_=29, *n*_HT_=34, *n*_CHD_=51) providing complete data.

### 2.2 Study design and procedure

All participants were instructed to abstain from caffeine and alcoholic beverages 24 h prior to study participation and to consume a semi-standardized breakfast before arriving for the baseline assessment at 8:00 h. Before resting study BP was assessed, participants completed questionnaires and had their height and weight measured to calculate the Body-Mass-Index (BMI). For the assessment of CHD risk factors (see below), blood samples were collected at 11:30 h, i.e., after fasting for 3.5 h since arrival. To assess longitudinal changes in CHD risk factors, all participants completed a follow-up assessment, including an identical blood sampling procedure, scheduled on average 3yrs later.

### 2.3 Biochemical analyses

CHD-risk was determined by measuring changes between baseline and follow-up assessment in the following biological risk factors: (1) coagulation activity by assessment of the prothrombotic factors D-dimer and fibrinogen, (2) pro-inflammatory activity by measurement of the cytokines IL-6 and TNF-α, as well as the acute phase protein CRP, (3) blood lipid profiles characterized by the ratio of total cholesterol (TC) to high-density lipoprotein cholesterol (HDL), and (4) HbA1c indicating average glucose levels over the previous 2–3 months, providing an indication of potential diabetes. Fibrinogen and D-dimer were analyzed at the Center for Laboratory Medicine of the Bern University Hospital (Inselgruppe AG, Bern) using standard quality procedures according to the Clauss method (31) for the determination of fibrinogen and a particle-enhanced immunoturbidimetric assay (INNOVANCE® D-Dimer, Siemens Healthcare GmbH, Erlangen, Germany) for the assessment of D-dimer levels. Blood lipids and HbA1c were analyzed in the same laboratory using *in vitro* assays for TC and HDL (enzymatic colorimetric, Roche, Mannheim, Germany) in addition to assays for the quantitative determination of HbA1c IFCC (mmol/mol) in whole blood (Tina-quant®, Roche, Mannheim, Germany). IL-6, TNF-α, and CRP were assayed in the laboratory of the Biological Work and Health Psychology group at the University of Konstanz. Cytokines were determined with a high sensitivity chemiluminescence sandwich immunoassay (Meso Scale Discovery, Rockville, USA), while CRP was determined using a high-sensitive enzyme immunoassay (ELISA, IBL Hamburg, Germany). For more details on the assays and missing data, see Supplementary Methods.

### 2.4 Psychological factors

#### 2.4.1 Optimism

Dispositional optimism was assessed by the corresponding subscale of the German version of the Revised Life Orientation Test (LOT-R; 32). Participants rated the extent to which they agreed with the three items “In uncertain times, I usually expect the best.”; “I am always optimistic about my future.”; “Overall, I expect more good things to happen to me than bad.” on a 5-point Likert scale (ranging from 0 = *strongly disagree* to 4 = *strongly agree*) yielding possible scores ranging from 0 to 12. The scale has sufficient psychometric properties (32) with an internal consistency of *α*=.72 in our sample.

#### 2.4.2 Positive and negative affect

Positive and negative affect were assessed by means of the validated German version of the Positive and Negative Affect Schedule (PANAS; 33). The PANAS consists of 20 items, 10 measuring positive affect (e.g., active, excited, strong) and 10 measuring negative affect (e.g., distressed, guilty, scared). All items are evaluated on a five-point Likert scale (ranging from 1 = *not at all* to 5 = *extremely*) to capture the degree to which the affect was experienced over the past 12 months. The items on the respective scales are averaged to obtain a single value (with possible scores ranging from 1 to 5) representing the degree of positive or negative affect. Higher values indicate a greater degree of positive or negative affect. Both scales have good psychometric properties (33) with internal consistencies of *α*=.87.

#### 2.4.3 Mindfulness

Mindfulness was measured using the German version of the Mindful Attention Awareness Scale (MAAS; 34). This scale evaluates the ability to attend to and remain aware of present life events and experiences (35). The scale comprises 15 items (e.g., “I run through activities without being really attentive”) to be rated on a 6-point Likert scale ranging from 1 = *almost always* to 6 = *almost never*. All items were reverse coded with a possible score range from 15 to 90, with high scores reflecting higher levels of mindfulness. Internal consistency was *α*=.92.

#### 2.4.4 Satisfaction with life

To assess global life satisfaction, participants completed the German version (36) of the Satisfaction with Life Scale (SWSL; 37). The scale comprises five items (e.g., “In most ways my life is close to the ideal”) which have to be rated on a 7-point Likert scale, ranging from 1 = *strongly disagree* to 7 = *strongly agree*, and possible scores ranging from 5 (low satisfaction) to 35 (high satisfaction). Cronbach´s *α* was. 80. For more information regarding missing questionnaire data, see Supplementary Material.

### 2.5 Statistical analyses

Statistical analyses were performed using SPSS (Version26.0) statistical software packages for MacIntosh (IBM SPSS Statistics, Chicago IL, USA). All tests were two-tailed with level of significance set at *p* < .05. Effect size parameters *f* and *R*^2^ changes are reported where appropriate (effect size conventions; small: *f* = .10, *ΔR*^2^=.02; medium: *f* = .25, *ΔR*^2^=.13; large: *f* = .40, *ΔR*^2^=.26) (38).

To compute an aggregated coagulation index for baseline coagulation activity, we averaged Z-transformed levels of D-dimer and fibrinogen. For an aggregated inflammatory index for baseline inflammatory markers, we accordingly averaged Z-transformed levels of IL-6, CRP, and TNF-α. Fibrinogen, D-dimer, TC/HDL-ratio, IL-6, CRP, TNF-α, and HbA1c changes from baseline to follow-up assessment were computed as differences between follow-up and baseline assessments. To compute an aggregated coagulation change index for changes in coagulation activity from baseline to follow-up, we averaged Z-transformed change levels of D-dimer and fibrinogen. For an aggregated inflammatory change index for changes in inflammatory markers from baseline to follow-up, we correspondingly averaged Z-transformed change levels of IL-6, CRP, and TNF-α.

All data were tested for normal distribution and homogeneity of variance using Kolmogorov–Smirnov and Levene's tests before statistical analyses. All measures showing a skewed distribution were log-transformed. While log-transformed data were used for modeling and testing, we depict untransformed data in Tables 1, 2, as well as in Supplementary Tabels S1 and S2 for reasons of clarity.

**Table 1:** Group characteristics and positive psychological factors at baseline (*N* = 200).

	*N* *=* *200*					*N* *=* *126*				
	CHD *n* = 86	HT *n* = 61	NT *n* = 53	*p*		CHD *n* = 55	HT *n* = 37	NT *n* = 34	*p*	
				HT, CHD, vs. NT	*HT vs. NT:* *CHD vs. NT:* *CHD vs. HT:*				NT, HT, vs. CHD	*HT vs. NT:* *CHD vs. NT:* *CHD vs. HT:*
Age [years]	64.76 ± 1.07 (37–85)	52.26 ± 1.56 (21–74)	49.26 ± 1.78 (25–78)	**<**.**001**	*HT vs. NT:.*19 *CHD vs. NT:* **<.001** *CHD vs. HT:* **<.001**	64.38 ± 1.33 (37–80)	52.81 ± 1.81 (31–74)	50.38 ± 2.3 (28–78)	**<**.**001**	*HT vs. NT:.*29 *CHD vs. NT:* **<.001** *CHD vs. HT:* **<.001**
BMI [kg/m^2^]	28.14 ± .43 (21.97–46.44)	28.40 ± .48 (20.35–38.86)	25.56 ± .42 (19.78–35.34)	**<**.**001**	*HT vs. NT:* **<.001** *CHD vs. NT:* **<.001** *CHD vs. HT:.*66	27.41 ± 0.42 (22.6–38.9)	28.65 ± 0.62 (20.35–38.86)	24.99 ± 0.54 (19.78–35.34)	**<**.**001**	*HT vs. NT:* **<.001** *CHD vs. NT:* **<.001** *CHD vs. HT:.*10
Study BP [mmHg]										
Study SBP	140.30 ± 1.66 (95.33–178.67)	150.95 ± 1.63 (120.67–188.00)	127.08 ± 1.17 (109.33–139.67)	**<**.**001**	*HT vs. NT:* **<.001** *CHD vs. NT:* **<.001** *CHD vs. HT:* **<.001**	139.05 ± 1.96 (95.33–172.33)	152.55 ± 2.19 (120.67–188)	127.15 ± 1.6 (109.33–139.67)	**<**.**001**	*HT vs. NT:* **<.001** *CHD vs. NT:* **<.001** *CHD vs. HT:* **<.001**
Study DBP	79.85 ± 1.08 (52.67–103.50)	93.19 ± 1.16 (72.67–113.67)	78.06 ± .94 (58.33–89.99)	**<**.**001**	*HT vs. NT:* **<.001** *CHD vs. NT:.*25 *CHD vs. HT_* **<.001**	81.05 ± 1.36 (52.67–103.5)	93.54 ± 1.62 (72.67–113.67)	78.03 ± 1.24 (58.33–89)	**<**.**001**	*HT vs. NT:* **<.001** *CHD vs. NT:.*13 *CHD vs. HT_* **<.001**
Home BP [mmHg]										
Home SBP		140.92 ± 1.36 (115.67–162.33) (*n* = 59)	124.07 ± 1.19 (105.17–148.33)	NT-HT: **<.001**			141.65 ± 1.73 (115.67–162.33) (*n* = 36)	123.78 ± 1.56 (105.17–148.33)	NT-HT: **<.001**	
Home DBP		84.63 ± .97 (68.33–100.83) (*n* = 59)	72.44 ± .84 (60.00–84.17)	NT-HT: **<.001**			85.77 ± 1.14 (74.83–100.83) (*n* = 36)	72.35 ± 1.01 (60–84.17)	NT-HT: **<.001**	
Medical characteristics										
Medication intake*	*n* = 86	*n* = 10				*n* = 55	*n* = 7			
LVEF ≤ 40% [%]	12 (14.0) (*n* = 84)					8 (14.5) (*n* = 53)				
MI [%]	50 (58.1) (*n* = 85)					29 (52.7) (*n* = 54)				
CABG [%]	24 (27.9) (*n* = 85)					14 (25.5) (*n* = 54)				
Smoking [%]	4 (4.7)					3 (5.5)				
Safety Lab (in HT only)										
Creatinine [*μ*mol/L]		80.74 ± 1.24 (64–103)					80.41 ± 1.46 (64–99)			
Sodium [mmol/L]		140.4 ± 0.28 (137–145) (*n* = 47)					140.29 ± 0.32 (137–144) (*n* = 28)			
Calcium [mmol/L]		2.36 ± 0.01 (2.11–2.58) (*n* = 47)					2.35 ± 0.02 (2.11–2.58) (*n* = 28)			
Potassium [mmol/L]		4.14 ± 0,04 (3.7–4.9) (*n* = 47)					4.11 ± 0,04 (3.7–4.6) (*n* = 28)			
Psychological factors										
Optimism	9.10 ± .21 (3.00–12.00)	9.16 ± .23 (2.00–12.00)	9.32 ± .25 (4.00–12.00)	.78		9.24 ± 0.25 (3–12)	9.46 ± 0.27 (7–12)	9.21 ± 0.33 (4–12)	.73	
Positive Affect	3.36 ± .07 (1.60–4.40)	3.43 ± .07 (2.40–4.60)	3.62 ± .07 (2.50–4.30)	.**028**	*HT vs. NT:*.**045** *CHD vs. NT:***.011** *CHD vs. HT:*.39	3.35 ± .08 (1.60–4.40)	3.50 ± .09 (2.50–4.60)	3.74 ± .08 (2.50–4.30)	.008	*HT vs. NT:*.**056** *CHD vs. NT:***.003** *CHD vs. HT:*.20
Negative Affect	1.66 ± .06 (1.00–3.70)	1.69 ± .08 (1.00–3.10)	1.62 ± .06 (1.00–2.90)	.97		1.64 ± .08 (1.00–3.70)	1.66 ± .09 (1.00–3.10)	1.65 ± .07 (1.10–2.90)	.91	
Mindfulness	69.22 ± 1.32 (41.00–90.00)	67.59 ± 1.41 (40.00–85.00)	66.63 ± 1.58 (36.00–90.00)	.48		68.93 ± 1.67 (41–90)	67.7 ± 1.89 (40–85)	67.56 ± 2.07 (36–90)	.88	
Satisfaction with life	26.95 ± . 45 (13.00–35.00)	28.02 ± .50 (13.00–35.00)	27.58 ± .38 (22.00–33.00)	.24		26.98 ± 0.58 (13–35)	28.14 ± 0.59 (20–35)	27.88 ± 0.47 (22–33)	.24	

Values are *M* ± *SEM*. CHD, CHD-patients; HT, hypertensive individuals; NT, normotensive individuals; BMI, body mass index; CABG, coronary artery bypass graft surgery; DBP, diastolic blood pressure; LVEF, left ventricular ejection fraction; MI, myocardial infarction; SBP, systolic blood pressure. *More details are provided in Supplementary Table S1. Statistically significant results are highlighted in bold.

**Table 2:** CHD risk factor at baseline and changes between baseline and follow-up in participants who completed both assessments.

	Total *N* = 126	CHD *n* = 55	HT *n* = 37	NT *n* = 34	*p*			
					HT, CHD, vs. NT	HT vs. NT	CHD vs. NT	CHD vs. HT
Time baseline to follow-up [months]	34.72 ± 0.62 (17–58)	32.47 ± 0.9 (17–58)	36.86 ± 0.95 (28–52)	36.03 ± 1.28 (26–51)	.002	.47	.020	<.001
Baseline coagulation index	1.79E-15 ± 0,07 (-1.10–4.4)	0.15 ± 0.13 (-1.1–4.4)	-0.10 ± 0.07 (-0.77–1.03)	-0.14 ± 0.11 (−1.05–1.87)	.16	.61	.11	.18
Coagulation change index	-1.56E-17 ± 0.07 (-2.94–2.32) (*n* = 124)	-0.08 ± 0.1 (-2.94–1.55) (*n* = 54)	0.10 ± 0.11 (-1.19–1.65) (*n* = 36)	0.02 ± 0.13 (-1.26–2.32)	.47	.57	.54	.23
Baseline fibrinogen [g/L]	2.65 ± 0.047 (1.65–4.46)	2.74 ± 0.08 (1.65–4.46)	2.63 ± 0.07 (1.98–3.58)	2.53 ± 0.09 (1.77–3.97)	.16	.37	.08	.29
Fibrinogen change [g/L]	0.09 ± 0.04 (-1.03–1.27) (*n* = 124)	0.13 ± 0.06 (-0.77–127) (*n* = 54)	0.07 ± 0.07 (-0.78–0.97) (*n* = 36)	0.05 ± 0.07 (-1.03–0.85)	.62	.81	.36	.51
Baseline D-Dimer [µg/L]	543.82 ± 57.7 (45–5177)	629.78 ± 124.05 (45–5177)	442.65 ± 34.78 (45–942)	514.85 ± 61.93 (45–1481)	.94	.73	.99	.76
D-Dimer change [µg/L]	296.02 ± 46.5 (-3678–2289) (*n* = 124)	163.33 ± 81.77 (-3678–1102) (*n* = 54)	426.67 ± 54.24 (0–1575)	368.42 ± 86.51 (-852–2289)	.17	.45	.24	.13
Baseline inflammation index	0.01 ± 0.06 (-0.86–3.18)	-0.04 ± 0.1 (-0.86–3.18)	0.06 ± 0.07 (-0.64–1.03)	0.03 ± 0.12 (-0.84–2.52)	.61	.69	.61	.32
Inflammation change index	-0.02 ± 0.07 (-3.14–4.10)	0.11 ± 0.12 (-1.34–4.1)	-0.05 ± 0.09 (-1–0.9)	-0.2 ± 0.15 (-3.14–1.45)	.15	.28	.08	.36
Baseline IL-6 [pg/mL]	0.64 ± 0.08 (0.03–8.30)	0.76 ± 0.16 (0.03–8.3)	0.57 ± 0.05 (0.16–1.48)	0.53 ± 0.1 (0.15–3.61)	.32	.12	.20	.96
IL-6 change [pg/mL]	0.09 ± 0.04 (-0.94–1.88)	0.14 ± 0.07 (-0.94–1.88)	0.05 ± 0.05 (-0.48–1)	0.08 ± 0.06 (-0.84–1.27)	.79	.81	.69	.52
Baseline TNF-α [pg/mL]	2.09 ± 0.06 (0.80–4.91)	2.05 ± 0.11 (0.8–4.42)	1.92 ± 0.07 (1.23–3.28)	2.32 ± 0.13 (1.12–4.91)	.043	.007	.046	.85
TNF-α change [pg/mL]	0.14 ± 0.06 (-3.39–3.23) (*n* = 124)	0.21 ± 0.08 (-1.04–3.23)	0.27 ± 0.08 (-0.36–2.31)	-0.12 ± 0.14 (-3.39–1.02) (*n* = 32)	.020	.019	.032	.56
Baseline CRP [μg/mL]	2.13 ± 0.18 (0.07–9.59) (*n* = 116)	1.72 ± 0.23 (0.07–6.96) (*n* = 53)	3.08 ± 0.35 (0.56–8.65) (*n* = 35)	1.71 ± 0.37 (0.11–9.59) (*n* = 28)	<.001	<.001	.95	<.001
CRP change [μg/mL]	0.58 ± 0.25 (-8.70–17.29) (*n* = 112)	1.11 ± 0.42 (-2.11–17.29) (*n* = 50)	0.01 ± 0.42 (-5.61–5.18) (*n* = 34)	0.32 ± 0.42 (-8.7–4.04) (*n* = 28)	.15	.88	.15	.032
Baseline TC/HDL	3.49 ± 0.09 (1.80–7.70)	3.13 ± 0.12 (1.8–7.7)	4.03 ± 0.15 (2.42–6.31)	3.49 ± 0.17 (2.01–5.49) (*n* = 32)	<.001	.013	.07	<.001
TC/HDL change	0.06 ± 0.07 (-2.30–2.74) (*n* = 116)	0.13 ± 0.12 (-2.3–2.74) (*n* = 49)	-0.09 ± 0.12 (-1.65–1.49) (*n* = 35)	0.1 ± 0.12 (-2.22–1.77)	.62	.36	.95	.40
Baseline HbA1c [mmol/mol]	37.58 ± 0.36 (26–48)	39.16 ± 0.49 (27–48)	36.46 ± 0.63 (28–43)	36.24 ± 0.68 (26–42)	<.001	.80	<.001	.001
HbA1c change [mmol/mol]	0.13 ± 0.51 (-7.00–38.00) (*n* = 104)	2.07 ± 1.16 (-6–38) (*n* = 41)	-1.03 ± 0.39 (-7–4) (*n* = 34)	-1.24 ± 0-44 (-6–3) (*n* = 29)	.003	.72	.005	.007

To compute group differences in subject characteristics and psychological factors at baseline (Table 1) as well as in CHD risk factors (Table 2) we used univariate ANOVAs.

To explore the potential clinical relevance of positive psychological factors for CHD risk, we tested whether positive psychological factors would relate to prospective changes in CHD risk factors. We calculated multivariate analyses of covariance (MANCOVA) with prospective changes in blood lipid profiles (TC/HDL-ratio), HbA1c as well as coagulation and inflammatory change indexes as dependent variables, applying listwise exclusion of missing dependent variables. As continuous independent variables, we entered optimism, positive affect, mindfulness, and satisfaction with life. To reduce model overfitting given our sample size (39), covariates were entered setwise as follows: in a minimally adjusted first model (1), we controlled for age at baseline, time between baseline and follow-up assessments, medication intake at baseline as well as changes in medication intake at follow-up, and study group. In a maximally adjusted second model(2), we additionally controlled for BMI at baseline, BMI change between baseline and follow-up, as well as negative affect. *post-hoc* testing of significant between-subject effects of the positive psychological factors on any dependent variables comprised linear regression analyses.

## 3 Results

### 3.1 Group characteristics

Table 1 provides a description of the demographic and medical characteristics as well as the positive psychological factors at baseline of CHD-patients, hypertensive and normotensive participants who participated in the first (*N* = 200) and both assessments (*N* = 126), respectively, (see Supplementary Material for detailed reasons for drop-out), with *N* = 104 providing complete data. The three groups differed in terms of age and BMI with CHD-patients being older (*p´s* < .001) and HT having a higher BMI compared to the other groups (*p´s* < .001). As expected, HT showed the highest systolic and diastolic BP compared with NT and CHD-patients (*p´s* < .001). On average, HT had serum levels of creatinine, calcium, sodium, and potassium in the normal reference range. In addition, all CHD-patients had medication (for more details, see Supplementary Table S1).

### 3.2 Positive psychological factors

Concerning the psychological factors, the three study groups differed in positive affect both in the first assessment [200: F(2,197) = 3.63, *p* = .028, *η*^2^*_p_* = .04, *f* = 0.20] and among participants who completed both assessments [126: F(2,123) = 4.97, *p* = .008, *η*^2^_p_ = .08, *f* = 0.29], with highest levels observed in NT and lowest levels found in CHD-patients. No group differences were evident for optimism, mindfulness, life satisfaction, and negative affect (*p´s* ≥.24; see Table 1).

### 3.3 CHD risk factors at baseline and follow-up

Table 2 shows CHD risk factors at baseline and changes from baseline to follow-up for the 126 participants who attended both assessments. The three groups differed in the time between the baseline and the follow-up assessment, with CHD-patients having the shortest time between assessments [*F*(2,123) = 6.47, *p* = .002, *η*^2^_p_ = .10, *f* = 0.33]. Hypertensives showed the highest CRP [*F*(2,113) = 8.95, *p* < .001, *η*^2^_p_ = .14, *f* = 0.40] and TC/HDL-ratio at baseline [*F*(2,123) = 11.64, *p* < .001, *η*^2^_p_ = .16, *f* = 0.43] as well as highest TNF-α changes between baseline and follow-up [*F*(2,121) = 4.05, *p* = .020, *η*^2^_p_ = .06, *f* = 0.25]. Normotentives had the highest baseline TNF-α levels [*F*(2,123) = 3.22, *p* = .043, *η*^2^_p_ = .05, *f* = 0.23]. CHD-patients showed highest HbA1c levels at baseline [*F*(2,123) = 8.16, *p* < .001, *η*^2^_p_ = .12, *f* = 0.37] and the greatest increases in HbA1c from baseline to follow-up [*F*(2,104) = 6.30, *p* = .003, *η*^2^_p_ = .11, *f* = 0.35].

Comparing follow-up participants (*n* = 126) to those who only completed the baseline assessment (*n* = 74), there were no differences in positive psychological factors or other baseline characteristics, except from higher baseline BMI [Follow-up: 27.12 ± 0.32(19.78–38.9); baseline only: 28.25 ± 0.49(21.97–46.44), *p* = .050], CRP (Follow-up: 2.13 ± 0.18[0.07–9.59; baseline only: 2.87 ± 0.28(0.31–11.55), *p* = .001], and HbA1c levels [Follow-up: 37.58 ± 0.36(26–48); baseline only: 39.62 ± 0.86(26–66), *p* = .029] in drop-out-participants.

### 3.4 Prediction of future CHD risk by positive psychological factors

#### 3.4.1 Positive affect

The main MANCOVA analysis (see Table 3 for an overview of the main longitudinal results) revealed that *positive affect* independently related to future overall CHD-risk [MANCOVA multivariate effects: (covariates model 1, Covariates-1: age at baseline, time between baseline and follow-up assessments, medication intake at baseline as well as changes in medication intake at follow-up, and study group) *F*(4,90) = 2.91, *p* = .026, *η*^2^_p_ = .11, *f* = 0.35, Wilk's*Λ*=.89); (covariates model 2, Covariates-2: additional control for BMI at baseline, BMI change between baseline and follow-up, as well as negative affect) *F*(4,87) = 2.81, *p* = .030, *η*^2^_p_ = .11, *f* = 0.35, Wilk's*Λ*=.89).

**Table 3:** Prediction of future CHD risk by positive psychological factors.

Independent variables	Minimally adjusted first model (1)	Maximally adjusted second model (2)
Positive affect		
Overall CHD-risk	*F*(4,90) = 2.91, *p* = .026, *η*^2^_p_ = .11, *f* = 0.35, Wilk's*Λ*=.89	*F*(4,87) = 2.81, *p* = .030, *η*^2^_p_ = .11, *f* = 0.35, Wilk's*Λ*=.89
Between-subject effects		
Coagulation change index	*F*(1,93) = 6.96, *p* = .010, η^2^_p_ = .07; *f* = 0.27/ß=-.31, *p* = .010, *ΔR*^2^ = 0.27	*F*(1,90) = 6.73, *p* = .011, η^2^_p_ = .07; *f* = 0.27/ß=-.31, *p* = .011, *ΔR*^2^ = 0.28
Post hoc fibrinogen	ß=-.37, *p* = .003, *ΔR*^2^ = 0.24	ß=-.36, *p* = .004, *ΔR*^2^ = 0.26
HbA1c	*F*(1,93) = 3.29,*p* = .073,η^2^_p_ = .03;*f* = 0.18	*F*(1,90) = 2.84, *p* = .096, η^2^_p_ = .03; *f* = 0.18
Satisfaction with Life		
Overall CHD-risk	*F*(4,90) = 2.92, *p* = .026, η^2^_p_ = .12, *f* = 0.37, Wilk's*Λ*=.89	*F*(4,87) = 3.07, *p* = .020, η^2^_p_ = .12, *f* = 0.37, Wilk's*Λ*=.88
Between-subject effects		
Inflammation change index	*F*(1,93) = 4.11, *p* = .045, η^2^_p_ = .04; *f* = 0.20/ß=-.27, *p* = .045, *ΔR*^2^ = 0.12	*F*(1,90) = 4.49, *p* = .037, η^2^_p_ = .05; *f* = 0.23/ ß=-.29, *p* = .037, *ΔR*^2^ = 0.15)
Post hoc IL-6	ß=-.38, *p* = .005, *ΔR*^2^ = 0.17	ß=-.39, *p* = .004, *ΔR*^2^ = 0.21;

Significant results of multivariate analyses of covariance (MANCOVA) with prospective changes in blood lipid profiles (TC/HDL-ratio), HbA1c as well as coagulation and inflammatory change indexes as dependent variables. Covariates were entered setwise as follows: in a minimally adjusted first model(1), we controlled for age at baseline, time between baseline and follow-up assessments, medication intake at baseline as well as changes in medication intake at follow-up, and study group. In a maximally adjusted second model(2), we additionally controlled for BMI at baseline, BMI change between baseline and follow-up, as well as negative affect.

**Figure 1:**
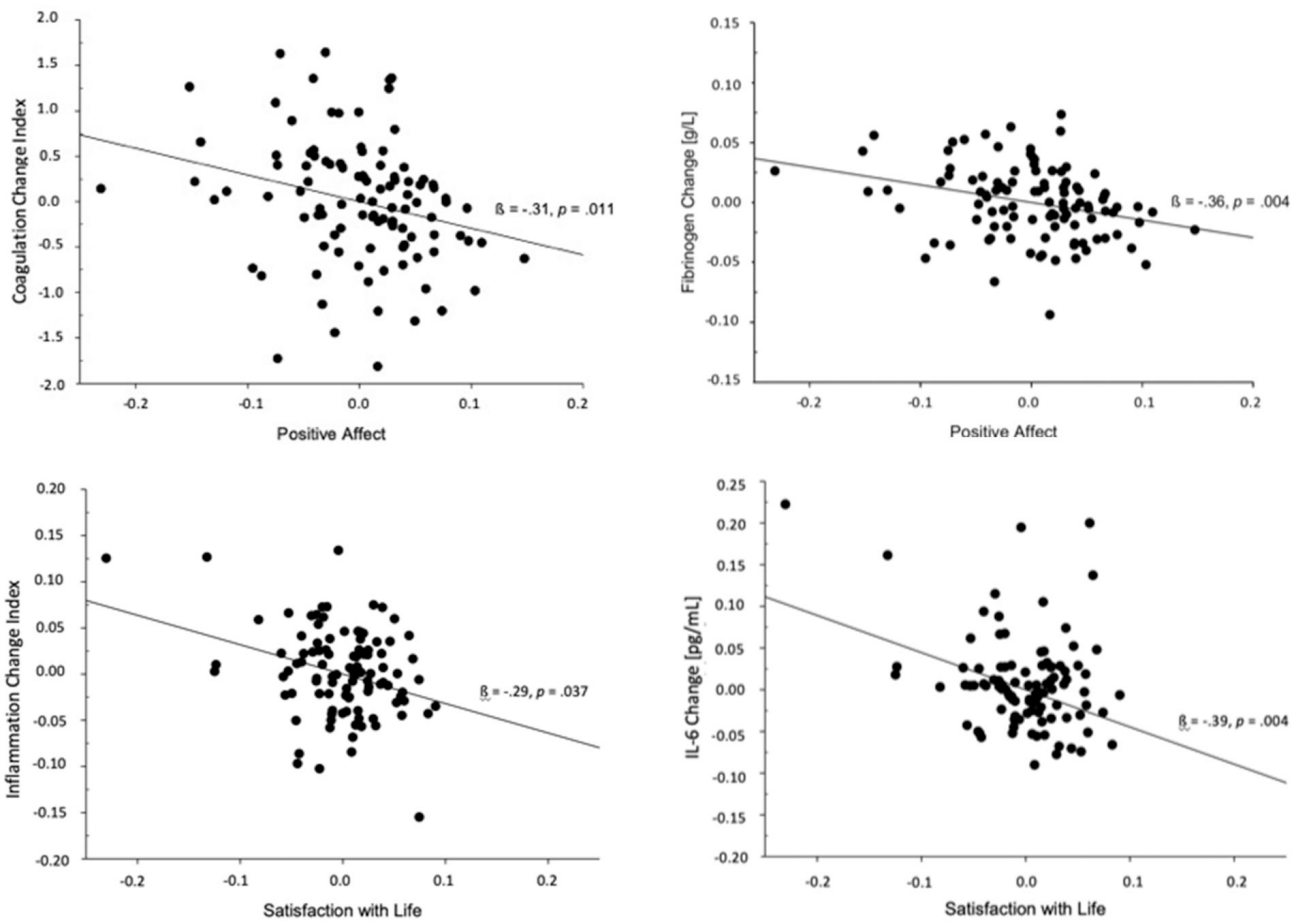
The figure depicts residuals of significant associations between positive psychological factors as independent variables and prospective changes in CHD risk factors as dependent variables controlled for the full set of covariates and positive psychological factors at baseline. Positive affect independently predicted **(A)** a lower coagulation change index (ß=-.31, *p* = .011, *ΔR*^2^ = 0.28) as well as **(B)** lower changes from baseline to follow-up in fibrinogen (ß=-.36, *p* = .004, *ΔR*^2^ = 0.26). Satisfaction with life independently predicted **(C)** lower increases in the inflammation change index (ß=-.29, *p* = .037, *ΔR*^2^ = 0.15) and IL-6 **(D)** (ß=-.39, *p* = .004, *ΔR*^2^ = 0.21) from baseline to follow-up.

In more detail, positive affect was significantly associated with lower increases in the coagulation change index (MANCOVA between-subject effects/*post-hoc* regression analysis: (Covariates-1) *F*(1,93) = 6.96, *p* = .010, *η*^2^_p_ = .07; *f* = 0.27/ß=-.31, *p* = .010, *ΔR*^2^ = 0.27; (Covariates-2) *F*(1,90) = 6.73, *p* = .011, *η*^2^_p_ = .07; *f* = 0.27/ß=-.31, *p* = .011, *ΔR*^2^ = 0.28). Further *post-hoc* testing revealed that positive affect related to lower increases from baseline to follow-up in fibrinogen [(Covariates-1) *ß*=-.37, *p* = .003, *ΔR*^2^ = 0.24; (Covariates-2) ß=-.36, *p* = .004, *ΔR*^2^ = 0.26; see Figures 1A,B], but not in D-dimer *(p´*s ≥ .38). Moreover, positive affect was borderline significantly associated with lower HbA1c increases from baseline to follow-up (MANCOVA between-subject effects: (Covariates-1) *F*(1,93) = 3.29, *p* = .073, *η*^2^_p_ = .03; *f* = 0.18; (Covariates-2) *F*(1,90) = 2.84, *p* = .096, *η*^2^_p_ = .03; *f* = 0.18; *post-hoc* regression analysis: (Covariates-1): ß=-.22, *p* = .073, *ΔR*^2^ = 0.22; (Covariates-2) ß=-.30, *p* = .096, *ΔR*^2^ = 0.29), but was neither related to the inflammatory change index (*p* ´s ≥ .40) nor to prospective changes in TC/HDL-ratio (*p* ´s ≥ .33).

#### 3.4.2 Satisfaction with life

Concerning *satisfaction with life*, MANCOVA analyses revealed an independent significant association with future overall CHD-risk [MANCOVA multivariate effects: (Covariates-1) *F*(4,90) = 2.92, *p* = .026, *η*2_p_ = .12, *f* = 0.37, Wilk's*Λ*=.89]; (Covariates-2) *F*(4,87) = 3.07, *p* = .020, *η*^2^_p_ = .12, *f* = 0.37, Wilk's*Λ*=.88). More precisely, satisfaction with life significantly related to lower increases in the inflammation change index (MANCOVA between-subject effects/*post-hoc* regression analysis: (Covariates-1) *F*(1,93) = 4.11, *p* = .045, *η*^2^_p_ = .04; *f* = 0.20/ß = -.27, *p* = .045, Δ*R*^2^ = 0.12; (Covariates-2) *F*(1,90) = 4.49, *p* = .037, *η*^2^_p_ = .05; *f* = 0.23/ ß=-.29, *p* = .037, *ΔR*^2^ = 0.15). Further *post-hoc* testing revealed associations with lower prospective changes in IL-6 [(Covariates-1) ß=-.38, *p* = .005, *ΔR*^2^ = 0.17; (Covariates-2) ß=-.39, *p* = .004, *ΔR*^2^ = 0.21; see Figures 1C,D], but not TNF-α (*p* ´s ≥ .27) or CRP (*p* ´s ≥ .49). Satisfaction with life was neither related to prospective changes in TC/HDL-ratio (*p* ´s ≥ .09), HbA1c (*p* ´s ≥ .55), nor to the coagulation change index (*p* ´s ≥ .13).

#### 3.4.3 Optimism and mindfulness

Neither *optimism* (*p* ´s ≥ .99) nor *mindfulness* (*p* ´s ≥ .30) were prospectively associated with CHD-risk.

Significant MANCOVA results were further confirmed by complementary *post-hoc* regression analyses using all available data for the respective dependent parameter (see Supplementary Material).

## 4 Discussion

We found differences in positive affect among the three groups, with the highest levels in normotensive individuals compared to lower levels in CHD-patients and hypertensives. Notably, these differences were independent of the other positive psychological factors under study (data not shown). Positive affect was lowest in CHD patients, who did not significantly differ from hypertensives. Although optimism, mindfulness, and life satisfaction were lowest, and negative affect was highest in CHD-patients, however without significant group differences in these factors. However, it needs to be considered that our study may not have been adequately powered to detect effects of small effect sizes.

Although no studies have yet compared positive psychological factors between healthy, diseased, and vulnerable groups, our results seem to align with findings indicating that low positive affect increases the risk of incident or recurrent CVD events (e.g., 5, 8). Notably, our HT being asymptomatic and not yet limited in daily life by the disease, did not differ from CHD-patients in positive affect levels. Therefore, our results suggest that low positive affect may increase disease risk rather than being solely a consequence of the advancing disease burden. Interestingly, we found associations with low levels of positive affect but none with negative affect, suggesting that insufficient positive affect seems to be the key factor here. Whether positive affect directly promotes cardiovascular health or serves as a buffer against adverse challenges (40) warrants further investigation.

We further found that both higher positive affect and life satisfaction were independently associated with reduced overall CHD-risk. In particular, higher positive affect predicted lower increases in coagulation (particularly fibrinogen levels). However, positive affect was not related to changes in inflammatory markers or TC/HDL-ratio. Higher life satisfaction predicted lower increases in inflammation (particularly in IL-6 levels) but was not related to prospective changes in TC/HDL-ratio, HbA1c, or coagulation. Neither optimism nor mindfulness were associated with prospective CHD-risk.

These results suggest that positive affect and life satisfaction have independent effects on CHD by influencing specific intermediate biological risk factors. Both, positive affect and satisfaction with life are essential components of hedonic well-being (41), which encompasses the emotional and cognitive aspects of happiness and pleasure (42, 43).

Hedonic well-being is discussed to improve cardiovascular health outcomes via different biological pathways (e.g 44): Previous research suggested that hedonic well-being may mitigate the impact of psychological stress on physiological responses either by promoting a more complete recovery (45) or by buffering the stress responses (46, 47). Moreover, well-being in general has been linked to lower basal cortisol levels (44, 47) and may facilitate parasympathetic activation, potentially reducing sympathetic nervous system responses (46). Furthermore, hedonic well-being has been shown to play a pivotal role in enhancing cardiovascular health by reducing (systemic) inflammation (44, 46, 48–50). Together, these pathways may help to explain the observed associations between satisfaction with life and reduced prospective increases in inflammation on the one hand and between positive affect and lower prospective increases in blood coagulation on the other hand. In more detail, chronic inflammation can lead to a prothrombotic state (51), which may underlie the observed effects on coagulation (and mainly fibrinogen increases). For further discussion, please see Supplementary Material.

We speculate that dispositional optimism and mindfulness might have limited effects on intermediate biological risk factors because both primarily involve cognitive processes and attitudes (11, 52) which may not be sufficient to trigger strong physiological mechanisms. Indeed, previous studies (e.g., 53, 54) have suggested that optimism and mindfulness alone might not activate stress-buffering pathways which could be critical for cardiovascular protection. While optimism and mindfulness may be helpful in terms of general mental (e.g., 11, 55) and cardiovascular health (56), their influence on physiological responses in acute situations such as e.g., heart rate variability (54) or attenuated cortisol stress reactivity (53), seems to be limited. Both, optimism and mindfulness may therefore not effectively counteract the physiological consequences of chronic stress, which include increases in intermediate biological risk factors for CHD (57).

We acknowledge several limitations of our study, including a relatively high drop-out rate and a broad follow-up range due to logistical constraints. Additionally, our sample size may have been insufficient to detect further group differences in the positive psychological factors (other than positive affect). Therefore, our findings should be replicated in larger samples. The generalizability to populations beyond middle-aged men of relatively high socioeconomic status remains uncertain, as previous studies suggest that the impact of positive psychological factors may vary by sociodemographic variables such as sex (41, 49) or socioeconomic status (58). Previous research suggests that positive psychological factors may exert particularly beneficial cardiovascular effects in men (41, 49). Accordingly, it is possible that the prospective associations observed in the present study would be weaker or even absent in women. Moreover, socioeconomic status may affect both positive psychological factors and cardiovascular risk through different mechanisms including lifestyle and stress-related pathways. Future studies should therefore take into account the effects of positive psychological factors on CVD risk in more diverse samples to clarify potential sex-specific effects and broader generalizability.

Although previous research suggests that life-style factors may mediate cardiovascular health effects of positive psychological factors (5, 6, 59, 60), our study design did not allow investigation of these potential mediating effects. Lifestyle factors and health behaviors were not assessed in the present study, as the primary focus was on positive psychological predictors and intermediate biological CHD risk factors. In addition, constraints related to the study duration and the participant burden associated with the longitudinal design further limited the inclusion of lifestyle-related assessments. This limits the ability to determine whether the observed associations are partially explained by health-related behaviors. Future studies should therefore include comprehensive assessments of lifestyle factors to clarify underlying mechanisms. Despite the study's findings, causality cannot be definitely established. Also, although controlled statistically, there were differences in time intervals between baseline and follow-up assessments and the medication use among CHD-patients and the small proportion of HTs. Medication may have mitigated CHD risk progression, possibly explaining the lack of significant associations between positive affect, life satisfaction, and lifestyle-related factors like blood lipids and HbA1c. Strengths of our study include adjustment for multiple confounders, such as negative affect(e.g., 61), consideration of the mutual effects of positive psychological factors, and a diverse sample encompassing healthy controls, HT, and CHD-patients, ensuring ample variability in biological risk factors.

Taken together, the findings of our study suggest that positive affect and satisfaction with life may contribute to maintaining and promoting cardiovascular health across individuals with varying levels of cardiovascular risk and disease burden. Also, hedonic well-being may represent a unique psychological domain that independently supports health and associated risk factors, rather than merely reflecting the absence of negative psychosocial factors (45, 62).

### Clinical perspectives

With respect to clinical implications, our study underscores the potential value of positive psychological interventions, particularly those that enhance positive affect and life satisfaction, in the prevention and treatment of CHD, as previously suggested (8, 63). Integrating strategies to increase positive affect and satisfaction with life may improve patient outcomes, enhance quality of life for patients and at-risk individuals, and potentially lower disease incidence in both at-risk and healthy populations.

## Data Availability

The raw data supporting the conclusions of this article will be made available by the authors, without undue reservation.

## References

[1] Tsao CW Aday AW Almarzooq ZI. Heart disease and stroke statistics—2023 update: a report from the American Heart Association. Circulation. (2023) 147:e93–e621. 10.1161/CIR.0000000000001123 36695182 PMC12135016

[2] Libby P Buring JE Badimon L. Atherosclerosis. Nat Rev Dis Primers. (2019) 5:56. 10.1038/s41572-019-0106-z 31420554

[3] Schmidt-Trucksäss A Lichtenstein AH von Känel R. Lifestyle factors as determinants of atherosclerotic cardiovascular health. Atherosclerosis. (2024) 395:117577. 10.1016/j.atherosclerosis.2024.117577 38852021

[4] Labarthe DR Kubzansky LD Boehm JK Lloyd-Jones DM Berry JD Seligman MEP. Positive cardiovascular health. J Am Coll Cardiol. (2016) 68:860–7. 10.1016/j.jacc.2016.03.608 27539179

[5] Levine GN Cohen BE Commodore-Mensah Y. Psychological health, well-being, and the mind-heart-body connection: a scientific statement from the American Heart Association. Circulation. (2021) 143:e763–83. 10.1161/CIR.0000000000000947 33486973

[6] Kubzansky Laura D Huffman Jeff C Boehm Julia K. Positive psychological well-being and cardiovascular disease. J Am Coll Cardiol. (2018) 72:1382–96. 10.1016/j.jacc.2018.07.042 30213332 PMC6289282

[7] Boehm JK Qureshi F Chen Y. Optimism and cardiovascular health: longitudinal findings from the coronary artery risk development in young adults study. Psychosom Med. (2020) 82:774–81. 10.1097/PSY.0000000000000855 32833896 PMC9901360

[8] Davidson KW Mostofsky E Whang W. Don't worry, be happy: positive affect and reduced 10-year incident coronary heart disease: the Canadian Nova Scotia health survey. Eur Heart J. (2010) 31:1065–70. 10.1093/eurheartj/ehp603 20164244 PMC2862179

[9] Boehm JK Peterson C Kivimaki M Kubzansky L. A prospective study of positive psychological well-being and coronary heart disease. Health Psychol. (2011) 30:259–67. 10.1037/a0023124 21553969 PMC3165195

[10] Boehm JK Peterson C Kivimaki M Kubzansky LD. Heart health when life is satisfying: evidence from the whitehall II cohort study. Eur Heart J. (2011) 32:2672–7. 10.1093/eurheartj/ehr203 21727096 PMC3205478

[11] Carver CS Scheier MF. Dispositional optimism. Trends Cogn Sci. (2014) 18:293–9. 10.1016/j.tics.2014.02.003 24630971 PMC4061570

[12] American Psychological Association (2018, April 19). APA Dictionary of Psychology. Available online at: https://dictionary.apa.org/life-satisfaction (Retrieved July 13, 2026).

[13] Loucks EB Britton WB Howe CJ Eaton CB Buka SL. Positive associations of dispositional mindfulness with cardiovascular health: the new England family study. Int J Behav Med. (2015) 22:540–50. 10.1007/s12529-014-9448-9 25339282 PMC4429005

[14] Hoen PW Denollet J de Jonge P Whooley MA. Positive affect and survival in patients with stable coronary heart disease: findings from the heart and soul study. J Clin Psychiatry. (2013) 74:14722. 10.4088/JCP.12m08022 23945449

[15] Yoo J Miyamoto Y Rigotti A Ryff CD. Linking positive affect to blood lipids: a cultural perspective. Psychol Sci. (2017) 28:1468–77. 10.1177/0956797617713309 28817363 PMC5633496

[16] Loucks EB Schuman-Olivier Z Britton WB. Mindfulness and cardiovascular disease risk: state of the evidence, plausible mechanisms, and theoretical framework. Curr Cardiol Rep. (2015) 17:112. 10.1007/s11886-015-0668-7 26482755 PMC4928628

[17] Figtree Gemma A Vernon Stephen T Harmer Jason A. Clinical pathway for coronary atherosclerosis in patients without conventional modifiable risk factors. J Am Coll Cardiol. (2023) 82:1343–59. 10.1016/j.jacc.2023.06.045 37730292 PMC10522922

[18] Roy B Diez-Roux AV Seeman T Ranjit N Shea S Cushman M. Association of optimism and pessimism with inflammation and hemostasis in the multi-ethnic study of atherosclerosis (MESA). Psychosom Med. (2010) 72:134–40. 10.1097/PSY.0b013e3181cb981b 20100888 PMC2842951

[19] Ikeda A Schwartz J Peters JL. Optimism in relation to inflammation and endothelial dysfunction in older men: the VA normative aging study. Psychosom Med. (2011) 73:664–71. 10.1097/PSY.0b013e3182312497 21949417

[20] Boehm JK Williams DR Rimm EB Ryff C Kubzansky LD. Relation between optimism and lipids in midlife. Am J Cardiol. (2013) 111:1425–31. 10.1016/j.amjcard.2013.01.292 23433765 PMC3644345

[21] Mohammadi N Aghayousefi A Nikrahan GR. The impact of an optimism training intervention on biological measures associated with cardiovascular health: data from a randomized controlled trial. Psychosom Med. (2020) 82:634–40. 10.1097/PSY.0000000000000834 32541548

[22] Shirom A Toker S Melamed S Berliner S Shapira I. Life and job satisfaction as predictors of the incidence of diabetes. Appl Psychol Health Well-Being. (2012) 4:31–48. 10.1111/j.1758-0854.2011.01054.x 26286969

[23] Boehm JK Chen Y Williams DR Ryff CD Kubzansky LD. Subjective well-being and cardiometabolic health: an 8–11 year study of midlife adults. J Psychosom Res. (2016) 85:1–8. 10.1016/j.jpsychores.2016.03.018 27212662 PMC4889157

[24] Uchino BN de Grey RGK Cronan S. Life satisfaction and inflammation in couples: an actor–partner analysis. J Behav Med. (2018) 41:22–30. 10.1007/s10865-017-9880-9 28884245 PMC5766426

[25] Brouwers C Mommersteeg PM Nyklíček I. Positive affect dimensions and their association with inflammatory biomarkers in patients with chronic heart failure. Biol Psychol. (2013) 92:220–6. 10.1016/j.biopsycho.2012.10.002 23085133

[26] Princip M von Känel R Sivakumar S. Longitudinal association between positive affect and blood lipids in patients following acute myocardial infarction. PLoS One. (2023) 18:e0287166. 10.1371/journal.pone.0287166 37917632 PMC10621864

[27] Degroote C von Känel R Thomas L. Lower diurnal HPA-axis activity in male hypertensive and coronary heart disease patients predicts future CHD risk. Front Endocrinol (Lausanne). (2023) 14:14. 10.3389/fendo.2023.1080938 PMC10036761 36967749

[28] Williams B Mancia G Spiering W. 2018 ESC/ESH guidelines for the management of arterial hypertension. Eur Heart J. (2018) 39:3021–104. 10.1093/eurheartj/ehy339 30165516

[29] Chalmers J MacMahon S Mancia G. 1999 World health organization-international society of hypertension guidelines for the management of hypertension. Guidelines sub-committee of the world health organization. Clin Exp Hypertens. (1999) 21:1009–60. 10.3109/10641969909061028 10423121

[30] Mancia G Kreutz R Brunström M. 2023 ESH guidelines for the management of arterial hypertension the task force for the management of arterial hypertension of the European society of hypertension: endorsed by the international society of hypertension (ISH) and the European renal association (ERA). J Hypertens. (2023) 41(12):1874–2071. 10.1097/HJH.0000000000003480 37345492

[31] Clauss A. Gerinnungsphysiologische schnellmethode zur bestimmung des fibrinogens. Acta Haematol. (1957) 17:237–46. 10.1159/000205234 13434757

[32] Glaesmer H Hoyer J Klotsche J Herzberg P. Die deutsche version des life-orientation-tests (LOT-R) zum dispositionellen optimismus und pessimismus. Zeitschrift für Gesundheitspsychologie. (2008) 16:1, 26–31. 10.1026/0943-8149.16.1.26

[33] Breyer B Bluemke M. Deutsche Version der Positive and Negative Affect Schedule PANAS (GESIS Panel). (2016).

[34] Michalak J Heidenreich T Ströhle G Nachtigall C. Die deutsche version der mindful attention and awareness scale (MAAS) psychometrische befunde zu einem achtsamkeitsfragebogen. Zeitschrift für Klinische Psychologie und Psychotherapie. (2008) 37:200–8. 10.1026/1616-3443.37.3.200

[35] Osman A Lamis DA Bagge CL Freedenthal S Barnes SM. The mindful attention awareness scale: further examination of dimensionality, reliability, and concurrent validity estimates. J Pers Assess. (2016) 98:189–99. 10.1080/00223891.2015.1095761 26560259

[36] Glaesmer H Grande G Braehler E Roth M. The German version of the satisfaction with life scale (SWLS). Eur J Psychol Assess. (2011) 27:127–32. 10.1027/1015-5759/a000058

[37] Diener E Emmons RA Larsen RJ Griffin S. The satisfaction with life scale. J Pers Assess. (1985) 49:71–5. 10.1207/s15327752jpa4901_13 16367493

[38] Cohen J. Statistical power analysis for the behavioral sciences. (1988).

[39] Babyak MA. What you see may not be what you get: a brief, nontechnical introduction to overfitting in regression-type models. Psychosom Med. (2004) 66:411–21. 10.1097/01.psy.0000127692.23278.a9 15184705

[40] Okely JA Weiss A Gale CR. The interaction between stress and positive affect in predicting mortality. J Psychosom Res. (2017) 100:53–60. 10.1016/j.jpsychores.2017.07.005 28789793 PMC5555349

[41] Zuccarella-Hackl C Princip M Sivakumar S von Känel R. Positive psychological well-being and cardiovascular health. Front Psychiatry. (2024) 15:15. 10.3389/fpsyt.2024.1443978 PMC11543404 39524133

[42] Ryan RM Deci EL. On happiness and human potentials: a review of research on hedonic and eudaimonic well-being. Annu Rev Psychol. (2001) 52:141–66. 10.1146/annurev.psych.52.1.141 11148302

[43] Diener E Suh EM Lucas RE Smith HL. Subjective well-being: three decades of progress. Psychol Bull. (1999) 125:276–302. 10.1037/0033-2909.125.2.276

[44] Dockray S Steptoe A. Positive affect and psychobiological processes. Neurosci Biobehav Rev. (2010) 35:69–75. 10.1016/j.neubiorev.2010.01.006 20097225 PMC2895001

[45] DuPont CM Weis TM Manuck SB Marsland AL Matthews KA Gianaros PJ. Does well-being associate with stress physiology? A systematic review and meta-analysis. Health Psychol. (2020) 39:879–90. 10.1037/hea0000979 32686951

[46] Pressman SD Cohen S. Does positive affect influence health? Psychol Bull. (2005) 131:925–71. 10.1037/0033-2909.131.6.925 16351329

[47] van Steenbergen H de Bruijn ERA van Duijvenvoorde ACK van Harmelen A-L. How positive affect buffers stress responses. Curr Opin Behav Sci. (2021) 39:153–60. 10.1016/j.cobeha.2021.03.014

[48] Blevins CL Sagui SJ Bennett JM. Inflammation and positive affect: examining the stress-buffering hypothesis with data from the national longitudinal study of adolescent to adult health. Brain Behav Immun. (2017) 61:21–6. 10.1016/j.bbi.2016.07.149 27444965

[49] Steptoe A Wardle J Marmot M. Positive affect and health-related neuroendocrine, cardiovascular, and inflammatory processes. Proc Natl Acad Sci U S A. (2005) 102:6508–12. 10.1073/pnas.0409174102 15840727 PMC1088362

[50] Steptoe A O'Donnell K Badrick E Kumari M Marmot M. Neuroendocrine and inflammatory factors associated with positive affect in healthy men and women: the whitehall II study. Am J Epidemiol. (2007) 167:96–102. 10.1093/aje/kwm252 17916595

[51] Esmon CT. Inflammation and thrombosis. J Thromb Haemostasis. (2003) 1:1343–8. 10.1046/j.1538-7836.2003.00261.x 12871267

[52] Bishop SR Lau M Shapiro S. Mindfulness: a proposed operational definition. Clin Psychol Sci Pract. (2004) 11:230–41. 10.1093/clipsy.bph077

[53] Endrighi R Hamer M Steptoe A. Associations of trait optimism with diurnal neuroendocrine activity, cortisol responses to mental stress, and subjective stress measures in healthy men and women. Psychosom Med. (2011) 73(8): 672–8. 10.1097/PSY.0b013e31822f9cd7 21949426

[54] Ede DE Walter FA Hughes JW. Exploring how trait mindfulness relates to perceived stress and cardiovascular reactivity. Int J Behav Med. (2020) 27:415–25. 10.1007/s12529-020-09871-y 32144687

[55] Coffey KA Hartman M Fredrickson BL. Deconstructing mindfulness and constructing mental health: understanding mindfulness and its mechanisms of action. Mindfulness (N Y). (2010) 1:235–53. 10.1007/s12671-010-0033-2

[56] Rozanski A Bavishi C Kubzansky LD Cohen R. Association of optimism with cardiovascular events and all-cause mortality: a systematic review and meta-analysis. JAMA Netw. Open. (2019) 2:e1912200. 10.1001/jamanetworkopen.2019.12200 31560385 PMC6777240

[57] Juster RP McEwen BS Lupien SJ. Allostatic load biomarkers of chronic stress and impact on health and cognition. Neurosci Biobehav Rev. (2010) 35:2–16. 10.1016/j.neubiorev.2009.10.002 19822172

[58] Boehm JK Kubzansky LD. The heart’s content: the association between positive psychological well-being and cardiovascular health. Psychol Bull. (2012) 138:655–91. 10.1037/a0027448 22506752

[59] Duque L Brown L Celano CM Healy B Huffman JC. Is it better to cultivate positive affect or optimism? Predicting improvements in medical adherence following a positive psychology intervention in patients with acute coronary syndrome. Gen Hosp Psychiatry. (2019) 61:125–9. 10.1016/j.genhosppsych.2019.06.001 31280918 PMC6861647

[60] Pressman SD Jenkins BN Moskowitz JT. Positive affect and health: what do we know and where next should we go? Annu Rev Psychol. (2019) 70:627–50. 10.1146/annurev-psych-010418-102955 30260746

[61] Chida Y Steptoe A. Positive psychological well-being and mortality: a quantitative review of prospective observational studies. Psychosom Med. (2008) 70:741–56. 10.1097/PSY.0b013e31818105ba 18725425

[62] Ryff CD Dienberg Love G Urry HL. Psychological well-being and ill-being: do they have distinct or mirrored biological correlates? Psychother Psychosom. (2006) 75:85–95. 10.1159/000090892 16508343

[63] Rozanski A Blumenthal JA Kaplan J. Impact of psychological factors on the pathogenesis of cardiovascular disease and implications for therapy. Circulation. (1999) 99:2192–217. 10.1161/01.CIR.99.16.2192 10217662

