# Supplementary material for: Longitudinal associations between positive psychological factors and cardiovascular health
Source: Front Cardiovasc Med. 2026 Jul 31;13:1821675. doi: 10.3389/fcvm.2026.1821675 (PMC13473298; doi:10.3389/fcvm.2026.1821675)
Supplement: Supplementary file 1 [file Supplementaryfile1.pdf]

Longitudinal Associations Between Positive Psychological Factors and Cardiovascular Health

Supplemental

Cathy Degroote[1], Roland von Känel[2], Claudia Zuccarella-Hackl[2,3], Livia Thomas[1,3], Hugo Saner[4], Roland Wiest[5] and Petra H. Wirtz[1,3,6*]

[1]Biological Work and Health Psychology, University of Konstanz, Konstanz, Germany

[2]Department of Consultation-Liaison Psychiatry and Psychosomatic Medicine, University Hospital Zurich, University of Zurich, Zurich, Switzerland

[3]Department of Psychology, University of Bern, Bern, Switzerland

[4]Institute for Social and Preventive Medicine, University of Bern, Bern, Switzerland

[5]Support Center of Advanced Neuroimaging, Institute of Diagnostic and Interventional Neuroradiology, University Hospital Bern, University of Bern, Bern, Switzerland

[6]Centre for the Advanced Study of Collective Behaviour, University of Konstanz, Konstanz, Germany

*CORRESPONDENCE:
Petra H. Wirtz
petra.wirtz@uni-konstanz.de

Supplemental Material

Study participants

Exclusion criteria for HT and NT. While there were no further exclusion criteria for CHD-patients, HT and NT were excluded if they engaged in regular strenuous exercise, reported alcohol or illicit drug abuse, atopic diathesis and allergies, chronic obstructive pulmonary disease, liver or renal diseases, rheumatic diseases, human immunodeficiency virus infection, cancer, psychiatric and neurological diseases, or a current infectious disease. These exclusion criteria were verified by a telephone interview that included a comprehensive health questionnaire (1,2). Non-patients (N=4) who reported regular medication intake stopped medication intake one week prior to the first study appointment. In order to increase the sample size of participants with hypertension, we accepted the use of antihypertensive medication in a small proportion of hypertensives (see Table-1 and Supplemental-Table-S1).

BP assessment. In a first step of the two-step assessment procedure, eligible individuals were asked to monitor their BP on three days by means of sphygmomanometer (Omron M6; Omron-Healthcare-Europe B.V., Hoofdorp, Netherlands). On these days, home BP was to be measured once in the morning and in the evening in a seated position after a 15-minute rest period. Participants were recruited as normotensive if the average home SBP was <135mmHg and the average home DBP was <85mmHg. In the second step, the preliminary assignment to NT or HT respectively was verified by three additional BP measurements (using Omron M6; Omron-Healthcare-Europe B.V., Hoofdorp, Netherlands) that were performed by trained personnel during the first study session, again in a seated position after a 15 minutes rest period. According to office BP cut-offs, hypertension was defined as mean study SBP of ≥140mmHg and/or mean study DBP of ≥90mmHg (3,4).

Reasons for drop-out at follow-up. At follow-up, included not reachable by phone (n=10), lack of time (n=19), no interest (n=11), excessive demand (n=3), severe illness (n=6), meanwhile living abroad (n=2), discontent with the study management (n=2), deceased (n=5) or no reason given (n=16). Notably, the study personnel in Bern was substantially reduced during the follow-up assessment due to organizational reasons (relocation of the working group from Bern to Konstanz). Consequently, the number of available follow-up time-slots per month was substantially reduced as compared to the baseline assessment, which led to a prolonged follow-up assessment period.

Missing data

For the questionnaires SWSL (n = 2) and MAAS (n = 3), 5 of the N = 200 participants at baseline had some missing items, that have been replaced by the respective scale average.

Due to technical problems sodium, potassium, and calcium could not be analyzed in 14 HT of the baseline study sample with 2 of them having a diagnosis of essential hypertension and being under antihypertensive medication.

For the N = 126 follow-up participants CRP could not be determined in 10 participants at baseline and in 8 participants at follow-up (see Table 2). As a consequence, for 12 participants, inflammation change indexes were computed as an index of IL-6 and TNF-α changes (without CRP). Moreover, in two further participants, TNF-α was also missing at follow-up. For these two participants the inflammation change index equals Z-transformed IL-6 changes from baseline to follow-up. In addition, at the follow-up assessment, fibrinogen and D-dimer clould not be determined in two participants. Moreover, TC/HDL could not be analysed in 10 and HbA1c in 22 participants rendering a total of N = 104 participants with complete data at follow-up. Reasons for missing data were problems with blood sampling or processing, and insufficient amount of sample for the respective analysis.

Assays for the prospective CHD risk assessment and HbA1c

Prothrombotic Measures. Venous blood was drawn into polypropylene tubes containing 3.8% sodium citrate (Sarstedt, Nümbrecht, Germany). Citrate tubes were immediately centrifuged for 20min at 4°C at 2000g and plasma was pipetted into aliquots. D-dimer was analyzed using a particle-enhanced immunoturbidimetric assay for the quantitative determination of D-dimers in human plasma (INNOVANCE® D-Dimer, Siemens Healthcare GmbH, Erlangen, Germany) on a Sysmex CS-5100 (Sysmex Europe, Norderstedt, Germany). Plasma fibrinogen levels were determined by a routine clotting assay applying standard quality procedures following the Clauss method. The intra- and inter-assay coefficients of variation were ≤7.9%.

Blood Lipids. We measured TotChol and HDL from heparine-coated monovettes (Sarstedt monovette orange). Analyses were performed using in vitro assays (enzymatic colorimetric) for the quantitative determination of blood lipids in human plasma (Roche, Mannheim, Germany) on a Roche/Hitachi Cobas C Analyzer (Roche, Mannheim, Germany). Mean inter- and intra-assay CVs were ≤1.2% and ≤2.5% respectively.

Pro-inflammatory Measures. For the determination of IL-6, TNF-α, and CRP venous blood was drawn in EDTA-coated monovettes (Sarstedt, Nümbrecht, Germany), and immediately centrifuged for 10min at 2000g and 4°C. Obtained plasma was stored at -80°C until analysis. IL-6 and TNF-α levels were determined with a high sensitivity chemiluminescence sandwich immunoassay (Meso Scale Discovery (MSD), Rockville, USA). CRP was determined using a high-sensitive enzyme immunoassay (ELISA, IBL Hamburg, Germany). For IL-6, mean inter- and intra-assay CVs were ≤7.3% and ≤4.5%. For TNF-α inter- and intra-assay CVs were ≤10.1% and ≤3.4% respectively. For CRP mean intra- and inter-assay CVs were <5.1% and 6.1% respectively.

HbA1c. To measure HbA1c, venous blood was drawn into EDTA-coated monovettes, and analyses were performed with in vitro assays for the quantitative determination of HbA1c IFCC (mmol/mol) in whole blood (Tina-quant®, Roche, Mannheim, Germany) using Roche/Hitachi Cobas C Systems (Roche, Mannheim, Germany). Mean inter- and intra-assay CVs were ≤1.6% and ≤2.0% respectively.

Supplemental Discussion

Higher positive affect predicted towards a trend level of significance lower increases in HbA1c levels. Our findings indicate that higher levels of positive affect are associated (borderline significantly) with lower increases in HbA1c levels. Given that blood glucose levels are strongly determined by diet and physical activity(5) this may suggest that lifestyle factors might influence the relationships between various aspects of hedonic well-being and cardiovascular health. In particular, positive affect has been shown to relate to healthier lifestyle choices, such as increased physical activity(6), or better adherence to medical advice(7). Together, these healthier habits may mediate the effect of hedonic well-being on intermediate biological CVD risk factors(8).

Supplemental Tables

Supplemental Tables S1

Medication

[%]	*N*=200		*N*=126	
	CHD *n* = 86	HT *n* = 10	CHD *n* = 55	HT *n* = 7
ACE inhibitors	32 (37.2)	4 (40.0)	18 (32.7)	2 (28.6)
AT2 antagonists	30 (34.9)	5 (50.0)	21 (38.2)	4 (57.1)
Anticoagulant	84 (97.7)	-	53 (96.4)	-
Alpha-blockers	6 (7.0)	1 (10.0)	3 (5.5)	1 (14.3)
Alpha-&beta-bl.	4 (4.7)	-	1 (1.8)	-
Beta-blockers	51 (59.3)	-	35 (63.6)	-
Antiarrhythmikum	4 (4.7)	-	2 (3.6)	-
Lipid-lowering drugs	80 (93.0)	-	51 (92.7)	-
Antidiabetikum	11 (12.8)	-	4 (7.3)	-
Insulin	-	-	-	-
Antiparkinson	3 (3.5)	-	2 (3.6)	-
Antidepressivum	6 (7.0)	-	3 (5.5)	-

Supplemental Table S2

Comparison of baseline group characteristics between participants completing follow-up assessment and drop-out participants.

	Follow-up (n = 126)	Drop-out (n = 74)	p
Age [years]	57.21±1.145 (28–80)	56.22±1.67 (21–85)	.47
BMI [kg/m^2]	27.12±0.32 (19.78–38.9)	28.25±0.49 (21.97–46.44)	.050
Study SBP [mmHg]	139.8±1.43 (95.33–188)	140.47±1.81 (112.5–178.67)	.77
Study DBP [mmHg]	83.9±1 (52.67–113.67)	82.66±1.25 (55–108)	.44
Fibrinogen [g/L]	2.65±0.05 (1.65–4.46)	2.75±0.08 (1.47–4.67)	.37
D-Dimer [µg/L]	543.82±57.7 (45–5177)	589.15±78 (45–4392)	.40
IL-6 [pg/mL]	0.64±0.08 (0.03–8.3)	0.58±0.04 (0.09–1.85)	.97
TNF-α [pg/mL]	2.09±0.06 (0.8–4.91)	1.95±0.09 (0.71–5.11)	.09
CRP [µg/mL]	2.13±0.18 (0.07–9.59) (n=116)	2.87±0.28 (0.31–11.55) (n=60)	.001
TC/HDL	3.49±0.09 (1.8–7.7)	3.58±0.11 (1.76–6.41)	.46
HbA1c [mmol/mol]	37.58±0.36 (26–48)	39.62±0.86 (26–66)	.029
Optimism	9.29±0.16 (3–12)	8.99±0.22 (2–12)	.25
Positive Affect	3.5±0.05 (1.6–4.6)	3.36±0.07 (1.7–4.4)	.11
Negative Affect	1.65±0.05 (1–3.7)	1.67±0.06 (1–2.9)	.86
Mindfulness	68.20±1.07 (36–90)	67.69±1.31 (38–90)	.86
Satisfaction with life	27.56±0.33 (13–35)	27.24±0.45 (13–33)	.57

Supplemental References

1. Zuccarella-Hackl C, von Känel R, Thomas L et al. Macrophage superoxide anion production in essential hypertension: Associations with biological and psychological cardiovascular risk factors. Psychosom Med 2016;78:750-757.
2. Zuccarella-Hackl C, von Känel R, Thomas L et al. Higher macrophage superoxide anion production in coronary artery disease (CAD) patients with Type D personality. Psychoneuroendocrinology 2016;68:186-193.
3. Chalmers J, MacMahon S, Mancia G et al. 1999 World Health Organization-International Society of Hypertension Guidelines for the management of hypertension. Guidelines sub-committee of the World Health Organization. Clin Exp Hypertens 1999;21:1009-60.
4. Mancia G, Kreutz R, Brunström M et al. 2023 ESH Guidelines for the management of arterial hypertension The Task Force for the management of arterial hypertension of the European Society of Hypertension: Endorsed by the International Society of Hypertension (ISH) and the European Renal Association (ERA). Journal of Hypertension 2023;41.
5. Colberg SR, Sigal RJ, Yardley JE et al. Physical Activity/Exercise and Diabetes: A Position Statement of the American Diabetes Association. Diabetes Care 2016;39:2065-2079.
6. Duque L, Brown L, Celano CM, Healy B, Huffman JC. Is it better to cultivate positive affect or optimism? Predicting improvements in medical adherence following a positive psychology intervention in patients with acute coronary syndrome. General Hospital Psychiatry 2019;61:125-129.
7. Ogedegbe GO, Boutin-Foster C, Wells MT et al. A Randomized Controlled Trial of Positive-Affect Intervention and Medication Adherence in Hypertensive African Americans. Archives of Internal Medicine 2012;172:322-326.
8. Huppert FA, Baylis N, Keverne B, Ryff CD, Singer BH, Dienberg Love G. Positive health: connecting well–being with biology. Philosophical Transactions of the Royal Society of London Series B: Biological Sciences 2004;359:1383-1394.
